# Hepatitis C virus‐free endoscope procedures project: An in‐hospital elimination approach

**DOI:** 10.1002/kjm2.12942

**Published:** 2025-01-16

**Authors:** Benjamin Lih‐Ren Hsieh, Yen‐Ting Kuo, Yu‐Ju Wei, Pei‐Chien Tsai, Ming‐Lun Yeh, Chung‐Feng Huang, Chia‐Yen Dai, Ming‐Yen Hsieh, Jee‐Fu Huang, Ming‐Lung Yu, Wan‐Long Chuang

**Affiliations:** ^1^ Hepatobiliary Division, Department of Internal Medicine Kaohsiung Medical University Hospital, Kaohsiung Medical University Kaohsiung Taiwan; ^2^ Department of Administration Kaohsiung Municipal Ta‐Tung Hospital, Kaohsiung Medical University Kaohsiung Taiwan; ^3^ Department of Internal Medicine Kaohsiung Municipal Ta‐Tung Hospital, Kaohsiung Medical University Kaohsiung Taiwan; ^4^ School of Medicine, College of Medicine, Kaohsiung Medical University Kaohsiung Taiwan; ^5^ Center of Excellence for Metabolic Associated Fatty Liver Disease, National Sun Yat‐sen University Kaohsiung Taiwan; ^6^ Hepatitis Center, Kaohsiung Medical University Hospital, Kaohsiung Medical University Kaohsiung Taiwan

**Keywords:** care cascades, elimination, endoscopy, hepatitis C virus, hospital safety

## Abstract

Hepatitis C virus (HCV) elimination in the care cascades for patients receiving invasive procedures remains elusive. This study aimed to evaluate the efficacy of HCV‐free Endoscope Procedures Project (CEPP) in the effort toward hospital HCV micro‐elimination in Taiwan. An electronic medical record (EMR)‐based remind system was introduced into gastrointestinal, surgical, urological, and gynecological departments prior to the endoscopy procedures. Anti‐HCV tests were actively ordered on their EMR among those patients who have not been tested in the past 5 years. Those patients with anti‐HCV+ were recruited into the care cascade for HCV treatment, including HCVRNA testing, direct antivirals (DAAs) delivery, and treatment response assessment. We divided the elimination project into two phases: before (2020 Jan to 2020 Dec, phase A) and during (2021 Jan to 2022 Sep, phase B) the remind system. The screening rate of phase B was 64.2% (1857/2893), which was significantly higher than phase A (18.7%, 899/4812) (*p* < 0.001). The screening rate of Department of Medicine (DOM) significantly increased from 21.1% of phase A to 89.3% of Phase B (*p* < 0.001). During phase B, the screening rate of non‐DOM was 48.2%, which was significantly higher than 11.8% of Phase A (*p* < 0.001). During Phase B, 15 (0.8%) out of 1857 screened patients were HCVRNA+. Six HCVRNA+ patients received DAAs treatment, and all achieved viral eradication. The CEPP significantly increased the anti‐HCV screening rate for subsequent care cascades, particularly in patients of DOM.

**Abbreviations**
HCV: hepatitis C virus
CEPP: HCV‐free Endoscope Procedures Project
EMR: electronic medical record
DAAs: direct antivirals
DOM: Department of Medicine
CHC: chronic hepatitis C
HCC: hepatocellular carcinoma
WHO: World Health Organization
HBV: hepatitis B virus
SVR: sustained virological response

## 1 INTRODUCTION

Hepatitis C virus (HCV) infection currently remains to be a global health threat, accounting for approximately 58 million infected patients and 290,000 deaths annually.^1^, ^2^ Chronic hepatitis C virus infection (CHC) may lead to liver cirrhosis and hepatocellular carcinoma (HCC). The invention of direct‐acting antivirals (DAAs) has reliably made the HCV cure possible in most CHC patients.^3^ However, CHC remains largely under‐diagnosed, presenting a barrier to patients receiving appropriate treatment. The asymptomatic characteristic during the early stages of CHC makes the exploration of the potential patients a challenging task.^4^ All of which may in turn lead to a reservoir for subsequent disease spreading and the emergence of hyper‐endemic areas.^5^, ^6^, ^7^, ^8^, ^9^ Therefore, it is crucial to establish procedures that can effectively identify more HCV patients eligible for effective treatment.

Recently efforts have been made in public prevention and therapeutic intervention for HCV to achieve the goal of hepatitis eliminate advocated by the World Health Organization (WHO) Global Health Sector Strategy on Hepatitis (2016–2021).^10^ Several milestones have been achieved regarding hepatitis prevention based on the considerations of patient safety and occupation safety for hospital staff. Our previous study demonstrated the significant efficacy of in‐hospital micro‐elimination project such as call back system or mandatory testing for cancer patient as well as increasing the HCV screening rate.^11^ The invasive procedures in hospital might carry the potential risk of HCV transmission. Endoscopic procedures are examples of the potential risk.^12^ Hence, preventing the risk of endoscopy‐related HCV transmission is still impactful in a clinical setting.

Consequently, we conducted the successive HCV‐free Endoscope Procedures Project (CEPP) aiming to evaluate the efficacy of the mandatory HCV screening for patients receiving endoscopy procedures. The screening rate before and during the in‐hospital micro‐elimination project were compared. We also compared the screening rates between procedures of different departments of the hospital.

## 2 MATERIALS AND METHODS

The Institutional Review Board of Kaohsiung Municipal Ta‐Tung Hospital/Kaohsiung Medical University Hospital approved this study before it was performed (Registered No. E‐20210009). The study was conducted in Kaohsiung Municipal Ta‐Tung Hospital, which is a regional referral hospital in Kaohsiung City, Taiwan. The profile of the hospital and the in‐hospital micro‐elimination project were depicted previously.^11^ The in‐hospital CEPP was prospectively implemented in January 2021. All patients receiving endoscopy procedures were recommended for anti‐HCV screening without anti‐HCV screening record on electronic medical record (EMR) within the past 5 years. The screening measurement was further noticed by a pop out reminding signal prior to the endoscopic procedures. We retrospectively collected the data before CEPP from 2020 January to 2020 December for comparison.

The screening rate of phase A (2020 January to 2020 December) and phase B (2021 January to 2022 September) was compared to evaluate the efficacy of the screening reminder system. Furthermore, the eligible CEPP patients were divided into those from Department of Medicine (DOM) and those who aren't (non‐DOM) to compare the screening rates between different departments.

### 2.1 Laboratory examination

We performed anti‐HCV tests with third‐generation commercially available enzyme‐linked immunosorbent assay kit (AxSYM 3.0; Abbott Laboratories, North Chicago, IL), with reactive samples tested in triplicate and confirmed via HCV RNA assay. We also used HCV reflex testing for patients who were anti‐HCV seropositive.^1^ We detected serum HCV RNA with a standardized, automated qualitative reverse‐transcription PCR assay (COBAS AMPLICOR Hepatitis C Virus Test, version 2.0; Roche, Branchburg, NJ). Previous tests were performed in duplicate with a detection limit of 50 IU/ml.

### 2.2 Statistical analysis

We used the Chi‐square test or Fisher's exact test to compare categorical variables of different groups. All statistical analyses were based on two‐sided hypothesis tests with a significance level of *p* < 0.05. Quality control procedures, database processing and analyses were performed using the SPSS 20.0 statistical package (SPSS, Inc., Chicago, IL).

## 3 RESULTS

The project was divided into two phases: before (2020 January to 2020 December, phase A) and during (2021 January to 2022 September, phase B) the screening electronic remind system. There were 8069, 3374, and 1290 patients who received gastrointestinal, surgical, and urological endoscope procedures in phase A, respectively. In phase B, the patients who received gastrointestinal, surgical, and urological endoscope procedures were 8976, 5652, and 2398, respectively. We examined the rate of screening, anti‐HCV (+), HCVRNA (+), treatment, and sustained virological response (SVR) for both phases.

### 3.1 Comparison between phase a and phase B

Phase A has a screening rate of 18.7% (899/4812), anti‐HCV+ rate of 4.0% (36/899), respectively. Four (11.1%, 4/36) HCVRNA+ patients were identified and three of them received DAA treatment. One patient with HCVRNA+ refused DAA treatment despite full‐scale explanation.

The screening rate and anti‐HCV+ rate in phase B was 64.2% (1857/2893), and 5.2% (97/1857), respectively. There were 15.4% (15/97) patients with HCVRNA+, and six of them received DAA treatment. Nine patients refused further DAA treatment despite explanation. All nine of the HCVRNA+ patients (3 of phase A and 6 of phase B) who received the DAA treatment have achieved sustainable virological response (SVR).

A total of 10 patients did not receive DAA treatment, including fear of adverse effects of anti‐viral treatment in four patients, economic concern in two patients, loss of follow‐up in two patients, old age in one patient, and one patient died before treatment, respectively.

**FIGURE 1:**
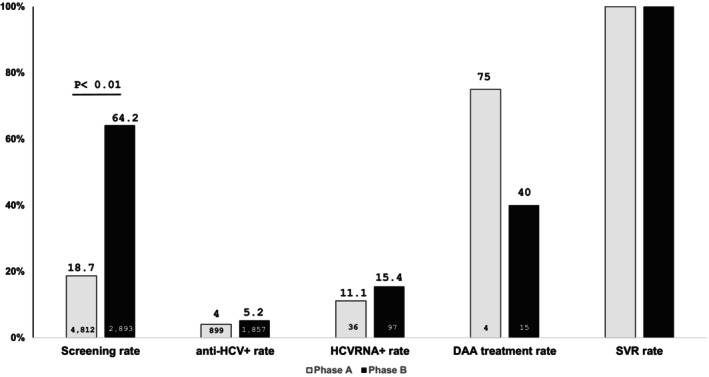
Care cascades in Phase A and Phase B.

The screening rate of Phase B was significantly higher than that of Phase A (64.2% vs. 18.7%, *p* < 0.01). There is no significant difference between the anti‐HCV+ rate and HCVRNA+ rate of both phases (Figure 1).

### 3.2 Comparison of DOM vs. non‐DOM

**FIGURE 2:**
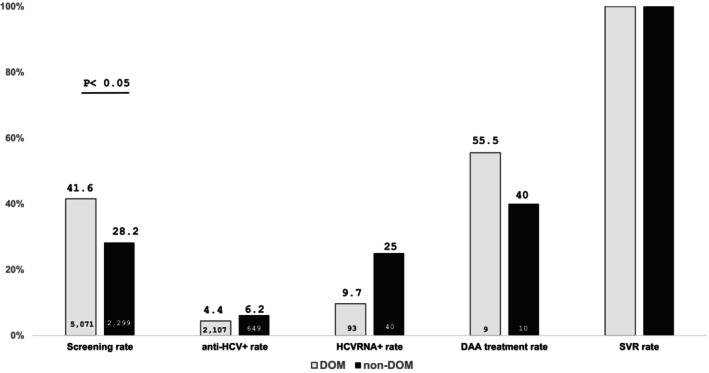
Care cascades in DOM and non‐DOM.

We divided all patients into two categories: patients from Department of Medicine (DOM) and non‐DOM. DOM patients of both phases have a screening rate of 41.6% (2107/5071), anti‐HCV+ rate of 4.4% (93/2107), HCVRNA+ rate of 9.7% (9/93), and a DAA treatment rate of 55.5% (5/9). On the other side, non‐DOM patients of both phases have a screening rate of 28.2% (649/2299), anti‐HCV+ rate of 6.2% (40/649), HCVRNA+ rate of 25.0% (10/40), and a DAA treatment rate of 40.0% (4/10). DOM patients had a significantly higher screening rate than non‐DOM patients (*p* < 0.05). All the patients who received DAA treatment have achieved SVR (Figure 2).

**FIGURE 3:**
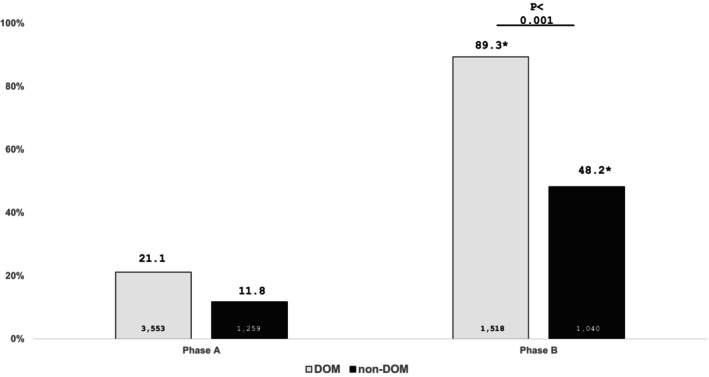
Screening rates of DOM and non‐DOM in Phase A and Phase B. *p < 0.001 compared to Phase A.

We also aimed to stratify the features of screening rate between DOM and non‐DOM according to different phases. The screening rate of DOM during phase A was 21.1% (751/3553), which significantly increased to 89.3% (1356/1518) during phase B (*p* < 0.001). During phase B, the screening rate of non‐DOM was 48.2% (501/1040), which was significantly higher than 11.8% (148/1259) of phase A (*p* < 0.001). The screening rate of DOM was significantly higher than non‐DOM (*p* < 0.001) (Figure 3).

## 4 DISCUSSION

In terms of occupational safety and patient safety, hospitals should be sterile and safe from infection to meet the health needs of all patients and staff. The study extended our previous efforts for in‐hospital HCV micro‐elimination.^11^ We demonstrated the EMR‐based screening remind system significantly increased the screening rate for patients receiving invasive endoscopy procedures. The successful HCV eradication could be readily achieved once the CHC patients were identified from the project. The efficacy of the system was more profound on DOM than non‐DOM. Our study provided the evidence of the feasibility of current HCV micro‐elimination project in a hospital level. The results could also pave the way for health policy‐making in a nation level.

Most efforts aiming to eliminate HCV have mainly focused on eliminating HCV infection in terms of disease burden, treatment costs, special populations, and community‐based outreach approaches. Hospital‐based elimination, mainly on the aspects of patient safety, staff occupational safety and infection control is a piece of work which can't be ignored. The issue of in‐hospital elimination is particularly important in Taiwan because iatrogenic factors are the major routes for HCV transmission.^13^, ^14^ The invasive characteristic of the endoscopic procedures is of significance in this view. Our study demonstrated that the prevalence of anti‐HCV+ was 4%–5.2% in this population, and a complete cure of HCV infection was observed for those HCVRNA+ patients upon identification by the project. Our results also shed a light on the expanding of the intervention into other non‐invasive procedures or into outpatient settings.

Meanwhile, a top‐down approach with EMR‐based screening remind system could significantly increase the anti‐HCV screening rate prior to endoscopic procedures. Su et al. implemented an electronic reminder system for anti‐HCV screening and HCVRNA testing in outpatient departments among diabetes patients. The results indicated that the total anti‐HCV screening rate increased from 49.3% to 78.2%, and the HCVRNA testing rate increased from 73.4% to 94.2%.^15^ Konerman et al. demonstrated that near all the newly diagnosed CHC patients were referred to specialty care with the increased HCV screening rate by using an EMR notification system within a short period.^16^ Our results were concordant with previous studies showing the efficacy of EMR alert in the significant increasing HCV screening rate. EMR‐based alert has been used as a measure for HCV micro‐elimination among baby boomer and immigrant.^16^, ^17^ These studies solidify the role of EMR alert in combating HCV. Further study is needed for the efficacy assessment of the similar alert system, potentially via multimedia, in increasing screening rate in a community level.

Simplifying the care cascades is an essential step toward HCV elimination. The increased treatment uptake was observed in our previous study which set up a reflex testing model for enhancing in‐hospital HCV elimination. The model included serial modalities such as HCV reflex testing, real‐time automatic appointments, and late call‐back for the missing patients. By implementing the model, HCV RNA diagnostic rate improves from 23.3% to 100%, and HCV treatment rate increased from 27.8% to 73.9% in non‐hepatology departments.^1^ The current study demonstrated that the screening rates of both DOM and non‐DOM increased significantly after the implementation of the project. DOM had a significantly higher screening rate than non‐DOM during Phase B. The comparison indicates that the impact of screening reminder system on the screening rate was better in DOM. Concordant results were shown in a study of HCV micro‐elimination project in diabetes patients. During that project, the implementation of electronical alert mainly increased the screening rates of nephrology and endocrinology departments, whereas the screening rates increase in other departments are overall less drastic.^15^ The possible explanations may include at least the followings: (1) Patients of DOM may have more scheduled visits than non‐DOM; (2) Most of patients of DOM received gastrointestinal visits and the gastrointestinal specialists were of much experience in terms of disease explanation, education, and treatment delivery; and (3) Care provides for patients of non‐DOM may focus on the disorders associated with the findings of endoscope procedures. To overcome the inequalities of disease awareness in both patients and care professionals in non‐DOM is a must for a comprehensive in‐hospital HCV micro‐elimination direction.

The current study had some limitations. First, some patients did not receive anti‐HCV screening prior to their endoscopic procedures since the EMR alert system was not mandatory. Therefore, a rescue program may be needed for the missed patients in this aspect, particularly in the non‐DOM. Second, we did not specifically investigate the reasons why clinicians or patients who did not respond to HCV screening tests for further analysis. To improve the disease awareness by continuous educational program in both primary care provider side and patient side could overcome the major hurdle in HCV care cascades. Third, the potential comorbidities of the patients were not recorded since the care cascades might be interfered with the patient factors. Lastly, the single‐center setting may limit generalizability and may have potential demographic biases. Future multicenter study would much enhance the study's scope.

In conclusion, the EMR‐based screening reminds project significantly increased the screening rate for patients receiving invasive endoscopy procedures. The screening rate significantly increased with the project implementation. Patients of DOM had a significantly higher increase in screening rate than those of non‐DOM. We therefore demonstrated a feasible model for in‐hospital HCV micro‐elimination in this special population.

## AUTHOR CONTRIBUTIONS

Study concept and design: Jee‐Fu Huang, Wan‐Long Chuang, Ming‐Lung Yu. Data collection and analysis: Benjamin Lih‐Ren Hsieh, Yen‐Ting Kuo, Jee‐Fu Huang, Ming‐Yen Hsieh. Statistical analysis: Yen‐Ting Kuo, Pei‐Chien Tsai, Jee‐Fu Huang. Draft of the manuscript: Benjamin Lih‐Ren Hsieh, Jee‐Fu Huang. Review and comment of the first version of the manuscript: Yu‐Ju Wei, Ming‐Lun Yeh, Chia‐Yen Dai, Chung‐Feng Huang, Ming‐Lung Yu. All authors read and approved the final manuscript.

## CONFLICT OF INTEREST STATEMENT

All authors declare that they have no conflicts of interest related to the study.

## ETHICS STATEMENT

The Institutional Review Board of Kaohsiung Municipal Ta‐Tung Hospital/Kaohsiung Medical University Hospital approved this study before it was performed (Registered No. E‐20210009). The study was performed in accordance with the ethical standards in the Helsinki Declaration of 1975, as revised in 2008.

## Data Availability

The data that support the findings of this study are available from the corresponding author upon reasonable request.
